# Development of an Evidence-Based Clinical Algorithm for Practice in Hypotonia Assessment: A Proposal

**DOI:** 10.2196/resprot.3581

**Published:** 2014-12-05

**Authors:** Pragashnie Naidoo

**Affiliations:** ^1^School of Health SciencesDiscipline of Occupational TherapyUniversity of KwaZulu NatalWestvilleSouth Africa

**Keywords:** hypotonia, clinical assessment, low muscle tone, evidenced-based

## Abstract

**Background:**

Assessing muscle tone in children is essential during the neurological assessment and is often essential in ensuring a more accurate diagnosis for appropriate management. While there have been advances in child neurology, there remains much contention around the subjectivity of the clinical assessment of hypotonia, which is often the first step in the diagnostic process.

**Objective:**

In response to this challenge, the objective of the study is to develop and validate a prototype of a decision making process in the form of a clinical algorithm that will guide clinicians during this assessment process.

**Methods:**

Design research within a pragmatic stance will be employed in this study. Multi-phase stages of assessment, prototyping and evaluation will occur. These will include processes that include a systematic review, processes of reflection and action as well as validation methods. Given the mixed methods nature of this study, use of NVIVO or ATLAS-ti will be used in the analysis of qualitative data and SPSS for quantitative data.

**Results:**

Initial results from the systematic review revealed a paucity of scientific literature that documented the objective assessment of hypotonia in children. The review identified the need for more studies with greater methodological rigor in order to determine best practice with respect to the methods used in the assessment of low muscle tone in the paediatric population.

**Conclusions:**

It is envisaged that this proposal will contribute to a more accurate clinical diagnosis of children with low muscle tone in the absence of a gold standard. We anticipate that the use of this tool will ultimately assist clinicians towards moving to evidenced based practice whilst upholding best practice in the care of children with hypotonia.

## Introduction

In recent years, the importance of early intervention and developmental programs has gained momentum and increased attention in order to promote general child health and well-being [1]. However, in order for early intervention to be optimally effective, early detection is paramount. As clinicians become progressively more involved in early examination, evaluation, and early intervention programs, the accuracy of these early examinations becomes essential in order to contribute to cogent decisions for intervention [2]. Accurate assessment and diagnosis is also essential in order to predict clinical course, manifestations, complications, prognosis, and to provide parental counselling.

Muscle tone assessment is an essential component during the neurological examination of infants and children. It is often crucial to the establishment of an accurate diagnosis and appropriate management of a child. A challenge for clinicians however, remains in the accurate identification and quantification of muscle tone due to the subjective nature of the clinical evaluation process [3]. This continues to confuse clinicians, and the scientific community has yet to gain consensus on the operational definition, the diagnostic criteria used to determine hypotonia, and the clinical assessment techniques for evaluation [4,5].

Hypotonia can be a confusing clinical presentation, often leading to inaccurate evaluation and unnecessary investigations due to the subjectivity of the assessment [6,7], which is frequently indicated in the literature [3-5,8-11]. If this confusion and uncertainty continues, it may have serious diagnostic implications (eg, psychological repercussions for both the child and family in terms of false labelling, invasive neurological investigations etc). Moreover, clinicians will continue to face the difficulty of accurately and consistently identifying children with hypotonia and diagnosis of the underlying cause. This may lead to a series of negative consequences with respect to proper management of these children and may also pose difficulty in conducting further research on the efficacy of intervention strategies or in the comparison of studies and an inability to apply or generalize results of studies to individuals.

There is evidence in the literature to indicate that the use of care pathways, algorithms, and practice guidelines, in clinical research will help in standardizing care and provide the necessary requirements for effective diagnostic and counselling interventions [12]. These guidelines and tools may also assist in providing a standard flowchart of the evidence-based diagnostic and treatment to be provided for a spectrum of diseases and disorders [12].

Although no gold standard currently exists, some researchers have begun to address the issue of criteria that may be used to assist in assessment of low muscle tone [4,5]. Thus, in order address the current challenges in clinical assessment of hypotonia, and in the move towards more evidence-based assessments, we will develop and validate a clinical algorithm for the assessment of hypotonia in children. The purpose of this tool would be to assist clinicians (paediatricians, occupational therapists, physiotherapists) in the decision making process in a stance towards more objective and accurate clinical diagnosis and making the appropriate referrals for early intervention. Such a study will also speak to the need for more evidence-based assessment and interventions as well as address the global health needs with respect to early detection and intervention. This will inevitably and indirectly advance the attainment of goal four, which is to reduce child mortality, of the United Nations Millennium Development goals [13].

## Methods

### Overview

This design research process follows the pragmatist paradigm. Above paradigmatic purism, the most appropriate approaches to the study are cross-stage mixed-methods. A three-phase approach will be used in this study.

### Phase 1 (Preliminary Phase): Identifying the Gaps and Analysis of the Problem

Phase 1 of the study will assist in the identification and analysis of the problem. We aim to undertake a systematic review to identify and appraise existing assessments that can be used by clinicians (occupational therapists, physiotherapists, and paediatricians) to detect hypotonicity in children. This will also assist in the identification of gaps that will further guide this research process.

Additional work indicated the need for more objective measures in the assessment of hypotonia [14]. A further study [15] gained consensus on the criteria, tests, and methods that would be most appropriate in determining whether a child has low muscle tone. These studies will be used in addition to the systematic review for phase 2 of the study.

### Phase 2 (Prototyping Phase): Development of the Clinical Algorithm

#### Overview

This phase of the study will involve the development and refinement of the clinical algorithm for the assessment of low muscle tone that is guided by theory, existing principles, and technology.

#### Instrument Development

The desktop approach in this phase will employ inductive, abductive, and deductive logic to the data from phase 1. This will develop from reflection upon experience (interpretivist), as well as from logical speculation from empirical data (positivist), in order to contribute towards the development of the clinical algorithm.

This process involves superimposition of the International Classification of Functioning (ICF) within the algorithm. The ICF also has merit in that it is a universal model that is holistic, based on human functioning not merely disability and is integrative, not merely medical or social. It is also operational and not driven by theory alone [16].

In order to ensure good practice for development of the algorithms, guidelines on the formal development of an algorithm will be considered. Formulation of evidence-based algorithms are said to be an increasing practice in both scientific papers and text books, however, their usefulness is questioned, as many of the authors are found not to adhere to the formal requirements [17,18]. Algorithms are said to be correct if formulated in accordance with technical regulations by the International Organization for Standardization (ISO) norm where each of the different symbols graphically represent the sequence for the solution to a problem [19]. The International Telecommunication Union (ITU-T) norm symbols that have been based on the ISO norms, have been incorporated approximately over a decade ago to adapt the algorithm for clinical practicability [17,19]. These will be considered in the technical aspects in the development of the algorithm in this study.

#### Instrument Refinement

This phase of the study will involve the refinement of the clinical algorithm for the assessment of low muscle tone with the interplay between reflection and action processes. Clinicians, opinion leaders, and clinical researchers who meet specified inclusion criteria will be solicited during conference workshop sessions as well as structured critique groups in order to implore feedback on the algorithm. We will employ nonprobability purposive sampling. The three clinical disciplines included in this study, will be considered a homogenous group as they belong to the same subculture or have similar characteristics [20]. Individuals will not be selected in an attempt to represent the general population, but rather in their ability to expertly contribute to the research process [21]. An iterative cycling process between phases of reflection and action will occur. Observation and recording of the process and outcomes of the workshop and structured sessions will occur, followed by reflection and refinement through process of modification, reframing, and rejection if necessary.

### Phase 3: Assessment and Validation

The methodological rigor with which the algorithm was developed will be subject to the critique of identified experts in the field with the use of the the Appraisal of Guidelines for Research & Evaluation II (AGREE-II) instrument developed by the AGREE Research Trust. The instrument is a generic tool designed primarily to help guideline developers and users assess the methodological quality of guidelines [22]. This tool consists of 23 key items organized within six domains followed by two global rating items. Each domain captures a unique dimension of guideline quality. All items are rated on a 7-point Likert scale. A quality score is calculated for each of the six AGREE-II domains. This will help access the rigour with which the algorithm was developed.

Additionally, we aim to establish epistemic correlation and content validity of the algorithm, via nonprobability purposive sampling of experts, which will include *inter alia,* international leaders in the field of paediatrics as highlighted by authorship in related articles identified in phase 1 (systematic review). A survey consisting of both open and closed ended questions will be developed after phase 2 of the study. Qualitative software packages such as NVIVO or Archiv fuer Technik, Lebenswelt und Alltagssprache-text interpretation (ATLAS-ti) will be used to code qualitative data and the Statistical Package for the Social Sciences (SPSS) version 21 will be used to quantify the responses on the survey. Both content analysis and descriptive statistics will be utilized to analyse the data. Further decisions related to analysis will be determined following the design of the questionnaire. If levels of agreement are necessary, Cronbach alpha will be computed and *a priori* thresholds for agreement will be set.

### Ethical Approval

The proposed study has received full ethical clearance from a human and social sciences ethics committee.

## Results

Initial results from the systematic review revealed a paucity of scientific literature that documented the objective assessment of hypotonia in children. The review identified the need for more studies with greater methodological rigor in order to determine best practice with respect to the methods used in the assessment of low muscle tone in the paediatric population.

## Discussion

This proposed study will be guided by the overarching principles of evidence-based practice and will contribute to instrument development, more specifically with respect to a clinical algorithm. The study will also assist in addressing the current gaps in the literature, which may be considered the epistemological contribution. The study will additionally inform and provide a multidisciplinary team (paediatricians, occupational therapists, physiotherapists) with a clinical tool that is based on evidence and validated. It will also provide knowledge into the best practices for the assessment of low muscle tone to address current diagnostic challenges, and finally will contribute empirical evidence towards shaping educational curricula in the absence of a gold standard.

## Multimedia Appendix 1

MRC Scholarship/Grant Letter.

## Multimedia Appendix 2

NRF Grant Award Letter.

## Abbreviations

AGREE-II: Appraisal of Guidelines for Research & Evaluation II
ATLAS-ti: Archiv fuer Technik, Lebenswelt und Alltagssprache-text interpretation
ICF: International Classification of Functioning
ISO: International Organization for Standardization
ITU-T: International Telecommunication Union
SPSS: Statistical Package for the Social Sciences

## References

[1] Blauw-Hospers CH, Hadders-Algra M (2005). A systematic review of the effects of early intervention on motor development. Dev Med Child Neurol.

[2] Pilon JM, Sadler GT, Bartlet DJ (2000). Relationship of Hypotonia and Joint Laxity to Motor Development During Infancy. Pediatric Physical Therapy.

[3] Bodensteiner JB (2008). The Evaluation of the Hypotonic Infant. Seminars in Pediatric Neurology.

[4] Martin K, Inman J, Kirschner A, Deming K, Gumbel R, Voelker L (2005). Characteristics of hypotonia in children: a consensus opinion of pediatric occupational and physical therapists. Pediatr Phys Ther.

[5] Martin K, Kaltenmark T, Lewallen A, Smith C, Yoshida A (2007). Clinical characteristics of hypotonia: a survey of pediatric physical and occupational therapists. Pediatr Phys Ther.

[6] Jan MMS (2007). The hypotonic infant: Clinical approach. Journal of Pediatric Neurology.

[7] Leyenaar J, Camfield P, Camfield C (2005). A schematic approach to hypotonia in infancy. Paediatr Child Health.

[8] Paro-Panjan D, Neubauer D (2004). Congenital hypotonia: is there an algorithm?. J Child Neurol.

[9] Harris SR (2008). Congenital hypotonia: clinical and developmental assessment. Developmental Medicine and Child Neurology.

[10] Laugel V, Cossée M, Matis J, de Saint-Martin A, Echaniz-Laguna A, Mandel JL, Astruc D, Fischbach M, Messer J (2008). Diagnostic approach to neonatal hypotonia: retrospective study on 144 neonates. Eur J Pediatr.

[11] Prasad AN, Prasad C (2011). Genetic evaluation of the floppy infant. Semin Fetal Neonatal Med.

[12] Miller VS, Delgado M, Iannaccone ST (1993). Neonatal hypotonia. Semin Neurol.

[13] United Nations: Goal 4: Reduce child mortality - Child deaths are falling, but not quickly enough to reach the target.

[14] Naidoo P (2013). Current Practices in the Assessment of hypotonia in children. South African Journal of Occupational Therapy.

[15] Naidoo P, Joubert RW (2013). Consensus on hypotonia via Delphi process. Indian J Pediatr.

[16] World Health Organization (2007). International Classification of Functioning, Disability, and Health: Children & Youth Version: ICF-CY. International Classification of Functioning, Disability, and Health: Children & Youth Version: ICF-CY.

[17] Khalil PN, Kleespies A, Angele MK, Thasler WE, Siebeck M, Bruns CJ, Mutschler W, Kanz KG (2011). The formal requirements of algorithms and their implications in clinical medicine and quality management. Langenbecks Arch Surg.

[18] Eitel F, Kanz KG, Hortig E, Tesche A (2000). Do we face a fourth paradigm shift in medicine--algorithms in education?. J Eval Clin Pract.

[19] Kanz KG, Eitel F, Waldner H, Schweiberer L (1994). [Development of clinical algorithms for quality assurance in management of multiple trauma]. Unfallchirurg.

[20] Holloway I, Holloway I, Wheeler S (2010). Qualitative Research in Nursing Healthcare.

[21] Skulmoski GJ, Hartman FT, Krahn J (2007). The Delphi Method for Graduate Research. Journal of Information Technology Education: Research.

[22] AGREE Collaboration (2003). Development and validation of an international appraisal instrument for assessing the quality of clinical practice guidelines: The AGREE project. Qual Saf Health Care.

