# Supplementary material for: Development of an Evidence-Based Clinical Algorithm for Practice in Hypotonia Assessment: A Proposal
Source: JMIR Res Protoc. 2014 Dec 5;3(4):e71. doi: 10.2196/resprot.3581 (PMC4275483; doi:10.2196/resprot.3581)
Supplement: Supplementary file 1 [file resprot_v3i4e71_app1.pdf]

SOUTH AFRICAN MEDICAL RESEARCH COUNCIL

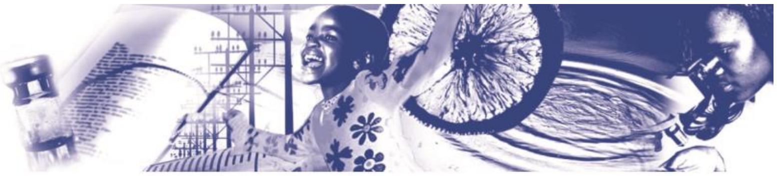

GRANTS AND SCHOLARSHIPS ADMINISTRATION
Sub Directorate

PO Box 09070, Tygerberg 7505, South Africa
Francie van Zijl Drive, Parow Valley, Cape Town
Tel: +27 (0)21 9380891; Fax: +27 (0)21 9380377

3 June 2014

To whom it may concern

RE: SCHOLARSHIP CONFIRMATION

This is to confirm that Pragashnie Naidoo, a PhD candidate at the University of KwaZulu-Natal holds a prestigious scholarship from the National Health Scholars Programme (NHSP). This was awarded in 2013 for the duration her PhD studies. The scholarship is a PhD development programme that is managed at South African Medical Research Council (MRC) under the Grants and Scholarships Administration Sub-Directorate. The MRC is one of South Africa's eight science councils whose main aim is to improve the nation's health status and quality of life through relevant and excellent health research.

In a highly competitive process, eligible South African health professionals are awarded a tax free scholarship for full-time study to either commence or complete a PhD degree. The level of funding is equivalent to the after-tax take-home pay of the candidate, based on the salary scales of the relevant post (or equivalent) in the National Department of Health. The value of the scholarship for Pragashnie Naidoo is **R289 196.52** per annum. The scholarship is renewable annually based on satisfactory progress.

Should further clarity be required on the scholarship or the conditions thereof, I will be delighted to be of assistance. Please do not hesitate to contact me on the details provided below.

Kind Regards,

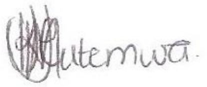

Dr M. Mutemwa

E-mail: Muyunda.Mutemwa@mrc.ac.za
Tel: +27 21 938 0945

Cc: Dr Thabi Maitin
Grants and Scholarships Administration Sub-Directorate
