# Supplementary material for: Development of an Evidence-Based Clinical Algorithm for Practice in Hypotonia Assessment: A Proposal
Source: JMIR Res Protoc. 2014 Dec 5;3(4):e71. doi: 10.2196/resprot.3581 (PMC4275483; doi:10.2196/resprot.3581)
Supplement: Supplementary file 2 [file resprot_v3i4e71_app2.pdf]

Contact : Sushie Pillay
Direct telephone : 012 481 4178
Direct fax : 086 697 2792
e-mail : ppillay@nrf.ac.za

Reference: TTK13070720688
UID: 88012
06 January 2014

Ms P Naidoo
Occupational Therapy
University of KwaZulu-Natal
naidoopg@ukzn.ac.za

Dear Ms Naidoo

APPLICATION FOR NRF GRANT FOR 2014: CSPG - THUTHUKA FUNDING INSTRUMENT ***(PhD Track)***

We have pleasure in informing you that your research proposal for funding from the above Programme has been accepted for support.

Short title: *Clinical Algorithm for the Assessment of Hypotonia in children*
Year of funding: 2014

The following table confirms your approved award for 2014:

Category	2014 NRF Award	2014 Institutional Commitment	2014 Total Award
**Student Support**			
Student Assistantships Summary	16,000.00	0.00	16,000.00
**Research Operating Costs**			
Running Expenses	27,500.00	27,500.00	55,000.00
Sabbatical/Study Visits	24,500.00	24,500.00	49,000.00
Research Equipment	1,500.00	1,500.00	3,000.00
**Total**	69,500.00	53,500.00	123,000.00

Running Costs consist of the following categories which have been totalled as indicated above:
- Research Materials and Supplies
- Domestic Travel (travel and subsistence)
- Conference Attendance: Local and Abroad
- Visiting Scientist/Mentoring
- *Ad hoc* Technical/Research Assistance and Lecturer Replacement
- External Supervisory Support
- Extended Study Visit (Abroad)

The funds towards Running Costs may be used within the requested budget items in your application and utilized in accordance with the rules as stipulated in the Thuthuka Programme 2014 Manual (available at https://nrfsubmission.nrf.ac.za Consult the Attached "Use and Management of Funds" for the day-to-day running expenses of the project.

GRANT CONDITIONS
This grant is subject to the attached Conditions of Grant.

RELEASE OF FUNDS
Funds for 2014 would only be released upon submission of the following documents via your Research Office:

1) The enclosed **'*Conditions of Grant for 2014*'** (*all pages*) must be submitted within one month of receipt of award letter **,** signed by yourself, the appropriate authority at your institution and witnesses (all pages preceding the last page must be initialled by all parties). Scanned copies are acceptable.
2) Reports outstanding for all grants received from any NRF Programme prior to 2014.
3) Grantholders in the PhD track are required to submit a proof of registration.

Failure to submit the above where applicable within one month of receipt of award letter may lead to the 2014 award being

cancelled.

GRANT STATEMENTS

Grantholders will be able to access grant statements on the NRF Online Submission system on a continuous basis. These statements generated at the end of each month, will provide a complete status of the grant in terms of amounts awarded, released, claimed (expensed) and paid per funding category. Statements can be accessed at https://nrfsubmission.nrf.ac.za through your 'Grantholder Tools' functionality.

It is important to note that payments will only be generated, once claims have been submitted directly to your institution's Finance Department, in order for your finance office to claim funds from the NRF.

GRANTHOLDER-LINKED STUDENT SUPPORT

Release of funds for grantholder-linked student support is subject to receipt of the on-line submission of nominations together with the required attachments at https://nrfsubmission.nrf.ac.za through "Grantholder Tools" functionality **BEFORE,**
31 March 2014 for full-year and 1st semester support. Nominations for **2nd semester support** must be submitted **before 9 August 2014.** Please take note of the contents of the enclosed *Conditions for the Award of Grantholder-linked Student Support Categories for 2014* regarding applicable criteria **BEFORE** completing the nomination.

The Agreement for Grantholder-linked Student Support must be signed by all signatories and included as an attachment to all online nominations.

PANEL FEEDBACK

Feedback from the review process is attached for your information and attention.

INSTITUTIONAL COMMITMENT

The Thuthuka Programme is based on a partnership between the Institution and the NRF. Your Institution has committed to a 1:1 contribution of this project (for Research Operating Costs only). The NRF will be undertaking an audit to verify the institutional contribution towards this project (as per Conditions of Grant).

REPORTING ON PROGRESS

Grantholders are required to submit an Annual Progress Report (APR) for continued funding in 2014 by**, 31 August 2014.** This APR should cover progress from the date of the award letter up to August 2014.

A Final Project Report is required for a previously funded, **completed,** Thuthuka project. The template is available from your Research Office.

CONTINUED FUNDING

Continued funding beyond 2014 will be based on the following information submitted in the APR:

- Significant **progress** on this project in line with the proposed objectives, substantiated in detail.
- Confirmation of research **activities for 2015**.
- **Well-motivated budget** for continued funding for 2015.

Continued funding will not be considered without this online APR.

A copy of this letter has been made available to your research administration.

We wish you well with your future research endeavours.

Yours sincerely

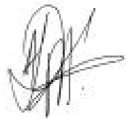

(Electronic signature)
Mrs Anthipi Pouris
Director: Capacity and Strategic Platforms Grants (CSP-G)
<u>Grants Management and Systems Administration</u>

Attachments
Conditions of Grant for 2014
Use and Management of Funds for 2014
Conditions for the Award of Grantholder-linked Student Support Categories for 2014
Agreement for Grantholder-linked Student Support
